# Research on Magnetic Field-Based Damage Detection Technology for Ferromagnetic Microwires

**DOI:** 10.3390/s24030878

**Published:** 2024-01-29

**Authors:** Haifei Wang, Junqing Yin, Cheng Xin, Chan Li, Yongdang Chen

**Affiliations:** School of Mechanical and Electrical Engineering, Xi’an Polytechnic University, Xi’an 710600, China; hfwang@stu.xpu.edu.cn (H.W.); chengx@stu.xpu.edu.cn (C.X.); chanli@stu.xpu.edu.cn (C.L.); chenyd@xpu.edu.cn (Y.C.)

**Keywords:** structural damage, ferromagnetic microwires, numerical calculation, health monitoring

## Abstract

Composite materials are frequently exposed to external factors during their operational service, resulting in internal structural damage which subsequently impacts their structural performance. This paper employs ferromagnetic materials for their sensitivity to magnetic field strength. By detecting variations in the magnetic field within the embedded ferromagnetic microwires of composite materials, the aim is to indirectly assess the health status of the composite materials. Firstly, a theoretical numerical model for magnetic field intensity at the crack site was established. Subsequently, a finite element model was employed to analyze the variations in the magnetic characteristics of ferromagnetic microwires at the crack site. Under different parameter conditions, the patterns of magnetic signals at the crack site were determined. The results indicate that with an increase in the angle between the external magnetic field and the crack, the fitted curve of the magnetic signal shows a linear increase. The distance between the peak and valley of the radial magnetic signal in the axial direction decreases, and the axial magnetic signal transitions from double-peak to single-peak. With the increase in crack depth, the fitted curve of the magnetic signal shows a linear increase, and the magnetic signal at the crack tip also exhibits a linear increase. An increase in crack width leads to a non-linear decrease in the fitted curve of the magnetic signal, and after reaching a certain width, the magnetic signal stabilizes. For two identical cracks at different distances, the magnetic signal exhibits a transition from a complete pattern to two complete patterns. With the increase in the external magnetic field, the magnetic signal shows a completely regular linear increase. By analyzing and calculating the variations in magnetic signals, the patterns of magnetic characteristics under the damaged state of ferromagnetic microwires were obtained. This serves as a basis for assessing whether they can continue in service and for evaluating the overall health status of composite materials.

## 1. Introduction

Composite materials find extensive applications in various fields such as aerospace, automotive, construction, and others. Due to their specific working environments, they are often subjected to various external factors such as temperature, chemical corrosion, and mechanical stress fatigue. This leads to the occurrence of microscopic damage in the material, which gradually develops until failure. The stress transfer from the failed part to adjacent fibers increases the damage level of nearby fibers, thereby affecting the overall structure [1,2,3]. During the in-service period of composite materials, the influence of the uncontrollable factors mentioned above often results in internal damage that is difficult to accurately detect through external means. This may lead to potential safety hazards. Therefore, the early detection and timely repair or replacement of damaged areas are crucial to ensuring safety. Health monitoring enables real-time tracking of the condition of composite materials, facilitating the early detection of potential damage and preventing degradation. This, in turn, contributes to prolonging the material’s lifespan and enhancing its safety. The most common factors leading to damage and failure in the internal structure of composite materials are internal defects. Common defects include fiber-matrix debonding, matrix cracking, fiber fracture, and so on [4,5,6]. In terms of fiber damage types in composite materials, cracks are typically the most detrimental failure type and a major cause of fiber fracture [7,8]. In the face of internal fiber damage, to effectively analyze damage within composite materials, it is essential to address a series of challenges, including the rational selection and arrangement of sensors, as well as the analysis and processing of data. Consequently, the rational detection of such damage has become a key research topic. A ferromagnetic microwire is a composite structure with a glass layer coating and a magnetic alloy core. It exhibits a unique giant magnetoimpedance effect [9,10]. Due to its small size, excellent mechanical properties, and rapid response, it serves as a soft magnetic material with high sensitivity to both magnetic fields and stress. Therefore, it can be employed as a magnetic sensor embedded in composite materials for applications in the field of health monitoring [11,12]. During the actual service process, when composite materials experience damage, the embedded ferromagnetic microwires closely adjacent to the fibers result in stress concentration in their vulnerable areas. This leads to the initiation and propagation of cracks, causing internal failure of the microfilaments within the matrix. As a consequence, they are unable to fulfill their intended role, rendering the ferromagnetic microwires ineffective in terms of monitoring damage to composite materials [13]. Therefore, analyzing and calculating the extent of damage at the cracks in iron magnetic microwires are of significant importance for detecting the health status of composite materials.

In research on composite material damage detection, non-contact methods such as ultrasonic, radiography, magnetic leakage, and penetration testing are commonly employed to penetrate the material and detect internal defects. However, the aforementioned methods pose some challenges when it comes to detecting internal fiber damage in composite materials. Ultrasonic and penetration testing exhibit limited sensitivity to fibers and penetration depth. Although radiographic testing can offer higher resolution, the radiation involved poses certain health hazards, necessitating caution in its application [14,15,16]. Furthermore, there are approaches utilizing deep learning methods for fault diagnosis in mechanical sensors for health monitoring purposes [17,18]. However, this paper primarily focuses on theoretical aspects and finite element analysis. Nonetheless, the environment during the actual service life of ferromagnetic microwires is complex, making it challenging to detect the extent of damage, especially given their inherent sensitivity to magnetic and mechanical properties. Therefore, this paper applies stress and a magnetic field for the unique stress concentration recognition and damage detection capabilities of ferromagnetic microwires. This allows for non-contact measurement to assess the degree of damage during the service life of ferromagnetic microwires [19,20]. The principle is that, upon magnetization of ferromagnetic materials, in order to achieve a stable state, the internal free energy of the material is minimized, leading to the reorganization of magnetic domains [21]. For defective ferromagnetic materials, applying and then removing stress results in residual stress remaining within the material. To counteract the increase in this portion of stress energy and achieve the lowest system energy, the reorganization of magnetic domains persists. This reorganization occurs in the form of magnetic charges accumulating on the surface of defects [22], generating a leakage magnetic field on the material’s defect surface. As a result, the magnetic field intensity becomes correlated with stress concentration and defect size. By detecting the magnetic signals and stress variations in the cracked region of ferromagnetic microwires embedded within composite materials, approximate information about the size, location, and potential re-initiation and propagation of cracks can be obtained [23,24]. This enables the assessment of whether the ferromagnetic microwires can continue to fulfill their role in health monitoring.

Ferromagnetic microwires consist of an outer glass layer and a core layer. Due to its unique structure and characteristics, existing research has indicated significant influences on the magnetic properties of the structure under different ratios of cladding to core [25], varying external temperatures [26], and diverse compositions of ferromagnetic microfilaments [27]. For ferromagnetic microfilaments with existing defects, the material properties of the Pyrex cladding [12], lacking magnetic characteristics, contrast with the decisive impact of the core, serving as the ferromagnetic material. Therefore, in the context of this paper, when defects occur in ferromagnetic microfilaments, only the influence of the core layer is considered. Under the action of external magnetic fields and stress, magnetic charges accumulate at the crack site, exhibiting changes in magnetic characteristics [28,29].

To further investigate the damage level of ferromagnetic microwires embedded in composite materials, this study establishes both a theoretical magnetic charge model and a finite element model for cracks in ferromagnetic microwires, followed by a comparative analysis of the results. By effectively analyzing the magnetic signal variation trends at the crack location in the radial, axial, and circumferential directions, this study reveals the magnetic signal changes at the crack under different parameter conditions. In conclusion, this method has successfully predicted the damage status of ferromagnetic microwires during service, providing a basis for determining whether they are suitable for the continued health monitoring of composite materials.

## 2. Numerical Model of Magnetic Charges for Cracks in Ferromagnetic Microwires

In terms of crack detection in ferromagnetic microwires, the orientation of the external magnetic field relative to the crack is random and uncertain. The relative direction between the crack and the magnetization field is a crucial factor influencing the magnetic signal detection. By applying an external magnetic field and external stress, the effective field *He* on the ferromagnetic microwires can be expressed as Equation (1) [30]:

(1) $$H_{e}=H+\alpha M+\frac{3\sigma }{2\mu_{0}}(\frac{d\omega }{dM})(\cos^{2}\theta -\nu^{2}\sin\theta )$$

In the equation, α is the coupling parameter, *H* is the applied magnetic field, *σ* is the externally applied stress, θ is the angle between stress and *H*, and *υ* is Poisson’s ratio. The vacuum permeability *μ*_0_ is 4π × 10^−7^ N/A, *M* is the magnetization intensity of the material, or the magnetic moment per unit volume. *ω* is the magnetostriction coefficient [31]. Expanding *ω* with the first two terms of the Taylor series yields:

(2) $$\omega =\gamma_{1}(\sigma )M^{2}+\gamma_{2}(\sigma )M^{4}$$

where *γ* is a constant to the order of one millionth. When the magnetic field *H* and stress *σ* change, the magnetization direction of the magnetic domains inside the ferromagnetic microwires will rotate toward the direction of stress. The magnetization intensity *M* at any point can be expressed as a function of the external magnetic field *H*:

(3) $$M=\chi_{m}H$$

In the equation, *χ_m_* represents the magnetization susceptibility (typically to the order of 10^3^–10^5^). Ferromagnetic materials can achieve a high magnetization intensity in a magnetic field, and the magnetization state is prone to saturation. After the rotation of magnetic domains, the magnetostrictive effect and the magnetomechanical effect constitute a mutually reversible process [32]:

(4) $${\left(\frac{d\omega }{dH}\right)}_{\sigma }={\left(\frac{dB}{d\sigma }\right)}_{H}$$

In the equation, (dω/dH)_σ_ represents the rate of change of the magnetostriction coefficient *ω* with respect to the magnetic field *H* under constant stress, and (dB/dσ)_H_ is the change in magnetic flux density *B* with respect to stress σ under constant magnetic field *H*. The relationship between magnetic field strength and magnetic induction intensity can be expressed by Equation (5):

(5) $${\left(\frac{dB}{d\sigma }\right)}_{H}=\mu_{0}{\left(\frac{dM}{d\sigma }\right)}_{H}$$

Magnetic field strength is used to indicate the direction and magnitude of the magnetic field, measured in Tesla (T), and is introduced in relation to the magnetic flux density at any point in the magnetic field. At this point, the relationship among internal magnetic hysteresis loss, magnetization intensity, and stress in the material is described in Equation (6):

(6) $$\frac{dM}{d\sigma }=\frac{\sigma }{\varepsilon^{2}}(M_{an}-M)+c\frac{dM_{an}}{d\sigma }$$

where *ε* and *c* are constants, and non-hysteresis magnetization intensity *M_an_* is represented as Equation (7):

(7) $$M_{an}=M_{s}\left(\coth(\frac{H_{e}}{a})-\frac{a}{H_{e}}\right)$$

where *M_s_* is the saturation magnetization intensity, and *a* is a constant. Due to the uneven distribution of stress concentration at the crack tip caused by the load on the ferromagnetic microwires, magnetic charge accumulates at the crack tip and is less distributed on both sides, forming an internal magnetic source. The uneven distribution of stress at the crack tip and on both sides leads to different magnetization intensities. The relationship between magnetic charge density *ρ* and external magnetic field *H* is shown in Equation (8) [23]:

(8) $$\rho =\chi_{m}\mu_{0}H$$

The distribution density of magnetic charge in ferromagnetic materials is closely related to the magnetization field intensity. Under the combined action of magnetic field and stress, the material becomes magnetized, accumulating a large amount of magnetic charge in the crack area. The direction between the crack and the external magnetic field is uncertain. We established a fixed coordinate system to describe the magnetic signals at the crack location of a cylindrical structure in 3D space. Taking a 90° V-shaped crack as an example, a stress-magnetic charge theoretical model is developed [33].

**Figure 1:**
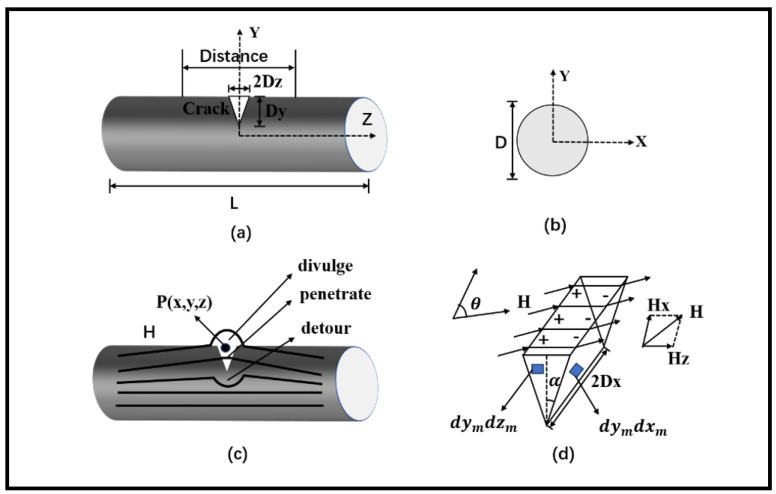
Schematic diagram of a typical crack model: (**a**) front view of the model, (**b**) side view of the model, (**c**) representation of magnetic signal principles, and (**d**) representation of the magnetic charge model.

The size and magnetic charge model diagram at the crack location of ferromagnetic microwires after cracking are shown in Figure 1. Here, L represents the length of the ferromagnetic microwires, D is its diameter, θ is the angle between the external magnetic field and the crack, and the external magnetic field *H* is parallel to the Z-axis in the positive direction. Distance reflects the size of the magnetic signal by taking points 50 μm on both sides of the center point of the upper surface of the crack along the Z-axis. α takes values between 0 and 90. A coordinate system is established in three orthogonal directions in the cracked area of the ferromagnetic microwires, with the length, width, and depth of the crack region being 2Dx, 2Dz, and Dy, respectively. The coordinates of the spatial point p are represented as (x, y, z), and the coordinates of the magnetic charge source point are (Xm, Ym, Zm) [23].

The principle of magnetic signal detection is that the magnetic field passing through the crack region forms a high magnetic reluctance zone. When magnetic lines pass through this zone, they are impeded, creating a magnetic potential difference in the crack sidewall region, as depicted in Figure 1c. In the penetration zone, according to the principle of the magnetic field passing through the interface between two media, when the magnetic field enters the air from the solid region, the magnetic lines are perpendicular to the crack sidewall. When entering the solid region from the crack’s air region, the direction is almost parallel to the external magnetic field. When re-entering the solid region from the crack’s air region, it advances almost along the edge. In the leakage area, most of the magnetic lines from the solid region entering the air region leak into the air. In the bypass area, similar to the principle of current conduction, magnetic lines must pass through areas with low magnetic reluctance, so they bypass the sharp corners of the crack [34,35].

With the stretching of the ferromagnetic microwires, damage occurs, leading to changes in the structure and internal stress. Cracks are generated, and stress concentration occurs in the crack region, forming a stress-damaged area. In the detection of ferromagnetic microwires, their magnetization state reaches saturation. The magnetic charge density distribution on both sides of the crack is relatively uniform, as shown in Figure 1d. The external magnetic field *H* can be decomposed into two orthogonal magnetization field directions along the X and Z axes. *H_X_* is perpendicular to both sides of the crack transversely, and *H_Z_* is perpendicular to both sides of the crack longitudinally. Therefore, the magnetic field can be decomposed into two components parallel to the X-axis and parallel to the Z-axis for analysis. Then, according to the principle of magnetic field superposition, the vector sum of the two components is calculated to obtain the magnetic field at the crack:

(9) $$H=H_{X}+H_{Z}$$

The surface magnetic charge densities in the crack stress concentration region are denoted as *ρ_X_* and *ρ_Z_*. The microelement on the magnetic core surface is *dymdzm* and *dymdxm*, and the magnetic field intensity generated at spatial point *p* can be expressed as Equation (10):

(10) $$\left\{\begin{array}{l} dH_{X}=\frac{\rho_{X}dy_{m}dz_{m}}{2\pi \mu_{0}{r_{1}}^{2}}\cdot \vec{r_{1}}\\ dH_{Z}=\frac{\rho_{Z}dy_{m}dx_{m}}{2\pi \mu_{0}{r_{2}}^{2}}\cdot \vec{r_{2}} \end{array}\right.$$

The distances *r*_1_ and *r*_2_ from the magnetic charge point *p* to the positive and negative magnetic charge lines, respectively, can be expressed as Equation (11):

(11) $$\left\{\begin{array}{l} r_{1}=\sqrt{{\left(x+x_{m}\right)}^{2}+{\left(y-y_{m}\right)}^{2}+{\left(z-z_{m}\right)}^{2}}\\ r_{2}=\sqrt{{\left(x-x_{m}\right)}^{2}+{\left(y-y_{m}\right)}^{2}+{\left(z-z_{m}\right)}^{2}} \end{array}\right.$$

The surface magnetic charge density *ρ_X_* and *ρ_Z_* on the crack surface are related to the relative magnetic permeability *μ_r_* of the material and the size of the crack. According to the principle of the magnetic dipole model, the magnetic charge density can be expressed as Equation (12):

(12) $$\left\{\begin{array}{l} \rho_{X}=5.3(\frac{D_{Y}/D_{X}+1}{D_{Y}/(D_{X}\mu_{r})+1})H_{X}\\ \rho_{Z}=5.3(\frac{D_{Y}/D_{Z}+1}{D_{Y}/(D_{X}\mu_{r})+1})H_{Z} \end{array}\right.$$

The crack is located on the X-axis at Xm, ranging from Dy to −Dy along the Y-axis, and from −Dz to Dz along the Z-axis. Performing a double integration over the crack surface yields the components of the magnetization field *H_X_* and *H_Z_* at the point P (x, y, z) along the three coordinate axes. The magnitudes of the radial, axial, and circumferential magnetic signals are expressed as Equations (13) and (14):

(13) $$\left\{\begin{array}{l} H_{X,x}=\frac{\rho_{X}}{4\pi \mu_{0}}\int_{-D_{y}}^{D_{y}}\int_{-D_{Z}}^{D_{Z}}\frac{xdy_{m}dz_{m}}{{\left[{\left(x-X_{m}\right)}^{2}+{\left(y-Y_{m}\right)}^{2}+{\left(z-Z_{m}\right)}^{2}\right]}^{3/2}}\\ H_{X,y}=\frac{\rho_{X}}{4\pi \mu_{0}}\int_{-D_{y}}^{D_{y}}\int_{-D_{Z}}^{D_{Z}}\frac{ydy_{m}dz_{m}}{{\left[{\left(x-X_{m}\right)}^{2}+{\left(y-Y_{m}\right)}^{2}+{\left(z-Z_{m}\right)}^{2}\right]}^{3/2}}\\ H_{X,z}=\frac{\rho_{X}}{4\pi \mu_{0}}\int_{-D_{y}}^{D_{y}}\int_{-Dx}^{D_{x}}\frac{(z-z_{m})dy_{m}dz_{m}}{{\left[{\left(x-X_{m}\right)}^{2}+{\left(y-Y_{m}\right)}^{2}+{\left(z-Z_{m}\right)}^{2}\right]}^{3/2}} \end{array}\right.$$

(14) $$\left\{\begin{array}{l} H_{Z,x}=\frac{\rho_{Z}}{4\pi \mu_{0}}\int_{-D_{y}}^{D_{y}}\int_{-D_{X}}^{D_{X}}\frac{zdx_{m}dy_{m}}{{\left[{\left(x-x_{m}\right)}^{2}+{\left(y-y_{m}\right)}^{2}+{\left(z-z_{m}\right)}^{2}\right]}^{3/2}}\\ H_{Z,y}=\frac{\rho_{Z}}{4\pi \mu_{0}}\int_{-D_{y}}^{D_{y}}\int_{-D_{X}}^{D_{X}}\frac{ydx_{m}dy_{m}}{{\left[{\left(x-x_{m}\right)}^{2}+{\left(y-y_{m}\right)}^{2}+{\left(z-z_{m}\right)}^{2}\right]}^{3/2}}\\ H_{Z,z}=\frac{\rho_{Z}}{4\pi \mu_{0}}\int_{-D_{y}}^{D_{y}}\int_{-Dx}^{D_{x}}\frac{zdx_{m}dy_{m}}{{\left[{\left(x-x_{m}\right)}^{2}+{\left(y-y_{m}\right)}^{2}+{\left(z-z_{m}\right)}^{2}\right]}^{3/2}} \end{array}\right.$$

Through the integration operations in Equations (13) and (14), the different magnetic signals in the X and Z-axis directions are calculated. At the spatial point P (x, y, z), according to the superposition principle, the magnetic field can be synthesized by combining the magnetic signal vectors in the X and Z-axis directions, resulting in the total magnetic signal in different directions at the crack location.

(15) $$H_{0}=H_{X_{0}}+H_{Z_{0}}$$

In the equation, *H_X_*_0_ represents the magnetic signal in the X direction, and *H_Z_*_0_ represents the magnetic signal along the Z axis. By modeling the magnetic dipoles in three-dimensional space and vector synthesis, it is possible to achieve the magnetic signal detection of ferromagnetic microwires in a cracked state.

## 3. Calculation of the Simulation

To accurately obtain the variation pattern of the magnetic signal at the crack of the ferromagnetic microwires, a force-magnetic coupling finite element model is established. The actual dimensions and material parameters of the ferromagnetic microwires are shown in Table 1 [36].

**Figure 2:**
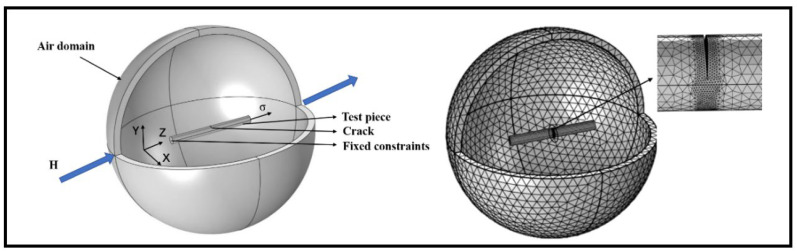
Model for magnetic signal detection and mesh partitioning.

**Figure 3:**
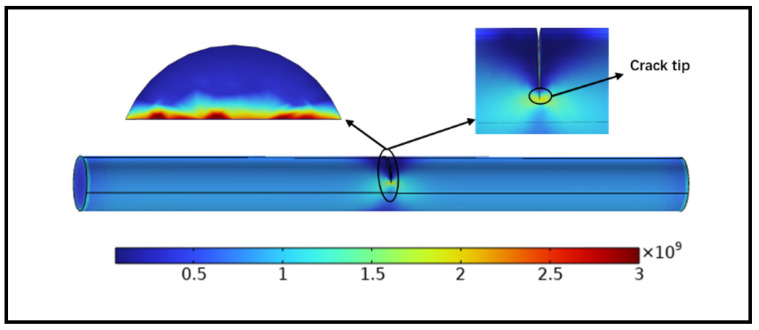
Stress plot of ferromagnetic microwires.

**Figure 4:**
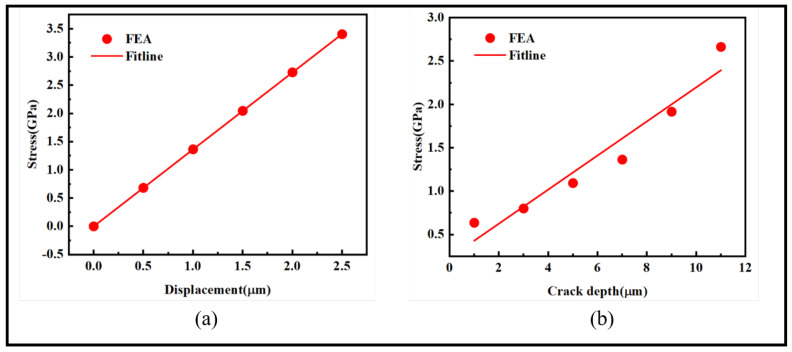
Stress distribution at the crack tip: (**a**) stress values under different tensile displacements and (**b**) stress values at different crack depths.

In finite element calculations involving the coupling of force and magnetism, multiple physical fields are considered, including the impact of air on the results. Therefore, it is necessary to incorporate an air domain to accurately simulate the interactions between physical fields. The length of the ferromagnetic microfilaments is chosen as 250 μm to ensure the accuracy of simulation results while considering computational resources. A spherical infinite air domain with a radius of 300 μm is established, and different mesh sizes are assigned to different domains. Mesh refinement is applied to the crack region to ensure result accuracy. One end of the specimen is subjected to fixed constraints, while a displacement along the Z-axis direction is applied to the other end. An external magnetic field of 80 A/m along the positive Z-axis direction is added. The solver is set with a step size of 0.5 μm. This completes the establishment of the magnetic signal detection model. The linear elastic solver is employed to ensure the accuracy of the calculation and expedite iterative convergence. To enhance the clarity of the model and result visualization, a V-shaped crack with θ set at 90°, a depth of 9 μm, and a width of 1 μm is chosen as an example to complete the model. The shape of the crack is illustrated in Figure 1d. As depicted in Figure 2 and Figure 3:

Figure 3 shows the stress distribution of the entire ferromagnetic microwire, the outer surface of the crack, and the two sides of the crack when subjected to a 1.5 μm displacement tensile load. As seen from the graph, stress is concentrated at the crack, especially at the crack tip, with darker colors indicating higher stress. The maximum stress value is 2.86 GPa. This indicates that the crack will first initiate and propagate at this location. The extracted stress values at the crack tip are shown in Figure 4.

As shown in Figure 4, in plot (a), the horizontal axis represents the stretching distance of the ferromagnetic microfilament, and the vertical axis represents the stress magnitude at the crack tip corresponding to the relative displacement. When the crack depth is 7 μm, with increasing displacement, the stress values at the crack tip linearly increase from 0 GPa at a tensile displacement of 0 μm to 3.404 GPa at a displacement of 2.5 μm, exceeding the maximum fracture stress and leading to crack initiation. In plot (b), the horizontal axis represents the crack depth, and the vertical axis corresponds to the stress values. The ferromagnetic microfilament is stretched by a fixed 1 μm. With an increase in crack depth, the stress values at the crack tip exhibit a linearly increasing trend, rising from 0.636 GPa at a crack depth of 1 μm to 2.663 GPa at a depth of 11 μm. The slopes of the fitted curves are 1.362 and 0.196, respectively, indicating that stress values increase with both tensile displacement and crack depth.

**Figure 5:**
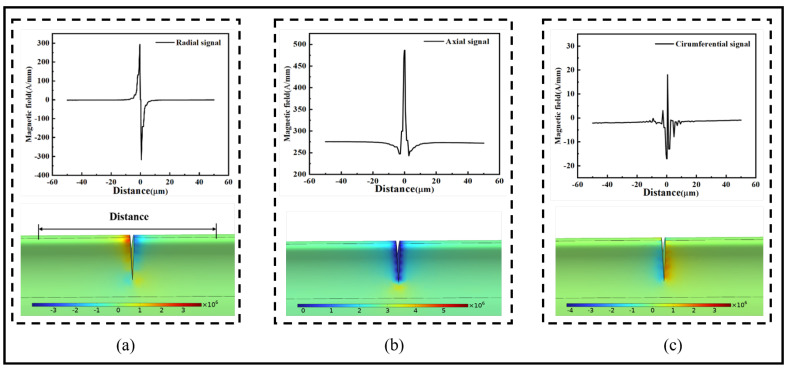
Comparison of magnetic signals and the model for a 90° crack: (**a**) radial magnetic signal, (**b**) axial magnetic signal, and (**c**) tangential magnetic signal.

Figure 5 shows a comparison between the model and the data for the radial, axial, and circumferential magnetic field components when the angle between the external magnetic field and the crack is 90°, respectively. The distance represents the magnetic signal along the surface of the crack on both sides of the ferromagnetic microwires, with a length of 50 μm. The radial magnetic signal of the 90° crack shows a trend of first increasing and then decreasing at the crack, reaching maximum values of 294.28 A/mm and minimum values of −316.76 A/mm at both ends of the crack tip. The axial magnetic signal at the crack exhibits a convex shape, with a maximum value reaching 486.44 A/mm. The circumferential component exhibits significant overall fluctuations at the crack, with a maximum value of 18.08 A/mm and a minimum value of −17.06 A/mm.

**Figure 6:**
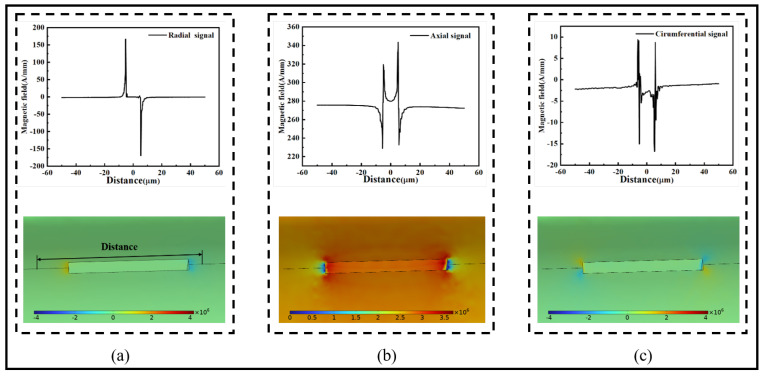
Comparison of magnetic signals and model for a 0° crack: (**a**) radial magnetic signal, (**b**) axial magnetic signal, and (**c**) tangential magnetic signal.

Figure 6 shows a comparison between the model and the data for the radial, axial, and circumferential magnetic field components. When the angle between the external magnetic field and the crack is 0°, the radial magnetic signal at the crack exhibits one peak and one trough, with a maximum value of 166.97 A/mm and a minimum value of −169.29 A/mm. The magnetic signal between the peaks is almost zero. The axial magnetic signal at the crack shows two peaks, 319.55 A/mm and 343.58 A/mm, and two troughs, 228.94 A/mm and 232.74 A/mm. The circumferential magnetic signal for the 0° crack also exhibits two peaks, 9.15 A/mm and 8.74 A/mm, and two troughs, −15 A/mm and −16.76 A/mm. However, the region between the peaks shows significant fluctuations. In summary, the circumferential magnetic signal exhibits significant fluctuations during propagation, while the radial and axial magnetic signals show distinct maximum and minimum values. The magnetic signal characteristics remain relatively stable during propagation, providing a more accurate representation of the variations in the impact of crack depth and width on the magnetic signal.

**Figure 7:**
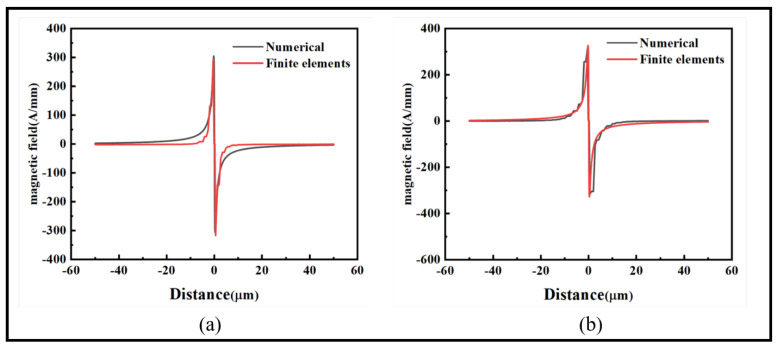
Comparison between finite element and numerical calculation: (**a**) comparison chart when the depth of the 90° crack is 3 μm, (**b**) comparison chart when the depth of the 90° crack is 7 μm.

The comparison charts in Figure 7 show the radial magnetic signal contrast between finite element simulations and numerical analyses based on the magnetic charge theory for a 90° crack with depths of 3 μm and 7 μm, both having a width of 1 μm. For the 3 μm depth, two peaks are observed at 296.28 A/mm and 305.78 A/mm, and two troughs at −313.76 A/mm and −305.03 A/mm. For the 7 μm depth, two peaks are observed at 318.68 A/mm and 327.15 A/mm, and two troughs at −318.89 A/mm and −327.07 A/mm. The relative error is around 3%, indicating a close match between the results obtained from the magnetic charge theory and finite element models, affirming the feasibility of calculating magnetic signals at the crack location in ferromagnetic microwires.

## 4. Results Analysis

1. **The impact of the angle θ between the external magnetic field and the crack on the magnetic signal**

**Figure 8:**
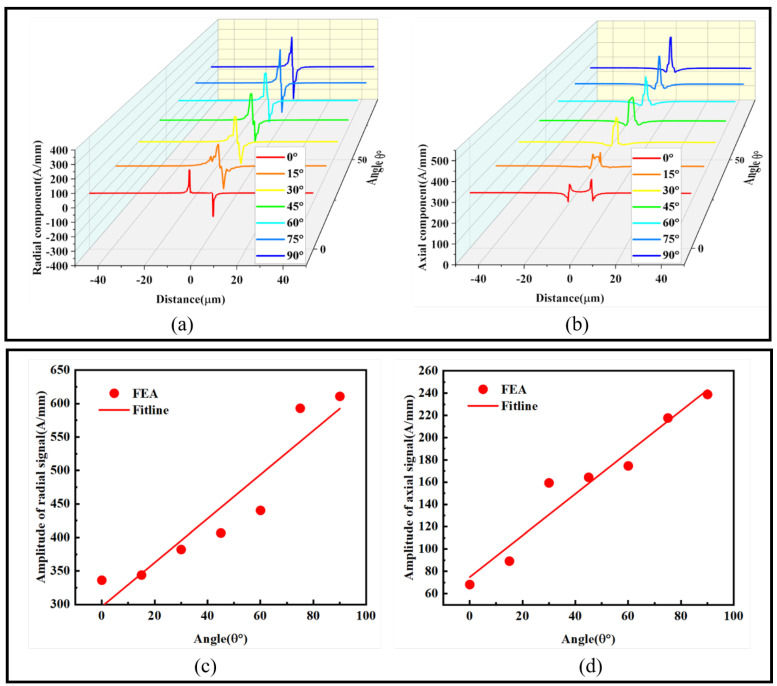
Influence of the angle θ on the magnetic signal: (**a**) radial magnetic signal, (**b**) axial magnetic signal, (**c**) radial magnetic signal amplitude difference fitting graph, and (**d**) axial magnetic signal amplitude difference fitting graph.

To investigate the influence of the angle θ between the external magnetic field and the crack on the magnetic signal, a crack with a depth of 3 μm and width of 1 μm was considered. The angle θ was varied from 0 to π/2, with a step of 15° for each test value. The amplitude of the fitted curve for the maximum and minimum values was obtained to represent the impact of θ on the radial and axial magnetic signals. The results are shown in Figure 8.

Figure 8a represents the variation of the radial magnetic signal with the increase in the angle θ. The peak increases from 166.97 A/mm to 294.28 A/mm, and the valley increases from −169.29 A/mm to −316.76 A/mm. Figure 8b represents the variation in the axial magnetic signal. The peak increases from 319.55 A/mm to 486.44 A/mm, and it transitions from two peaks to a single peak. This is due to the fact that at 0 degrees, the distance between peaks on the Z-axis is 2Dx. As the angle increases, the intersection between the magnetic signal line and the crack becomes shorter, changing from 2Dx to 2Dz, resulting in a shorter distance between peaks. Figure 8c shows that the radial magnetic signal amplitude fitting curve increases linearly. In Figure 8d, the axial magnetic signal amplitude fitting curve also exhibits a linear increasing trend. The slopes of the fitting curves are 3.29 and 1.87, respectively, demonstrating that the magnetic signal at the crack site increases with the angle. The fitting curves are capable of predicting the deviation angle of cracks in ferromagnetic microwires during service.

2. **Influence of Crack Depth Dy on the Magnetic Signal**

**Figure 9:**
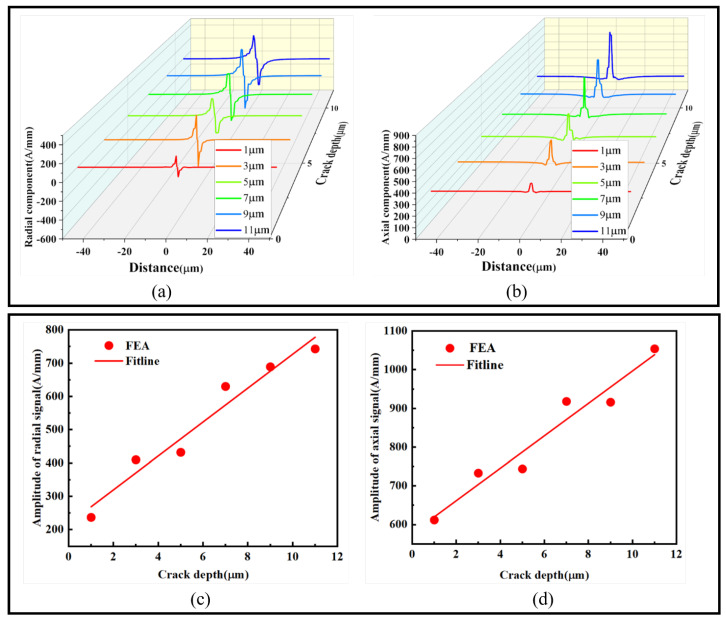
Influence of crack depth on the magnetic signals: (**a**) radial magnetic signal, (**b**) axial magnetic signal, (**c**) radial magnetic signal amplitude difference fitting graph, and (**d**) axial magnetic signal amplitude difference fitting graph.

To study the influence of crack depth on axial and radial magnetic signals, a crack with a width of 1 μm and θ = 90° was set, and the depth varied from 1 to 11 μm with a 2 μm interval. The maximum and minimum values were extracted to fit curves representing the impact of crack depth on radial and axial magnetic signals. As shown in Figure 9:

Figure 9a shows that the radial magnetic signal increases with the depth, with the peak increasing from 126.83 A/mm to 354.48 A/mm, and the trough increasing from −126.83 A/mm to −389.82 A/mm. Figure 9c presents the fitted curve of the radial magnetic signal, showing a linear upward trend. Figure 9b shows that the axial magnetic signal’s peak increases from 347.83 A/mm to 813.9 A/mm, while the trough fluctuates between 233.99 A/mm and 260.51 A/mm. Figure 9d presents the fitted curve of the axial magnetic signal, displaying a linear upward trend. The slopes of the fitted curves are 50.9 and 41.9, respectively, indicating that with the increase in crack depth, both radial and axial magnetic signals fluctuate significantly, and the magnetic signals increase linearly at a fast rate. Thus, analyzing the numerical changes in magnetic signals effectively predicts the depth of the crack.

**Figure 10:**
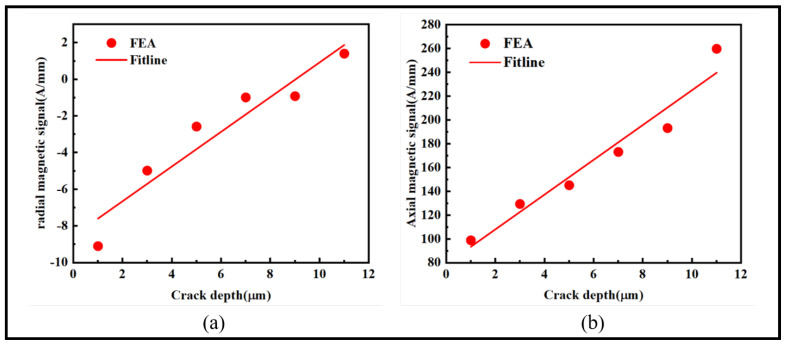
Illustration of the magnetic signals at the crack tip for different depths: (**a**) radial magnetic signal and (**b**) axial magnetic signal.

When subjected to stress, ferromagnetic microwires develop damage zones at the crack, with crack initiation occurring at the tip. This process can lead to structural failure. Therefore, by monitoring the changes in magnetic signals at the crack tip, it becomes possible to effectively predict whether the crack will undergo further expansion. The variations in radial and axial magnetic signals at the crack tip, along with the fitting curves at different points, are depicted in Figure 10:

Based on the relationship between the radial and axial magnetic signals at the crack tip and the crack depth as shown in Figure 10, we established fitting curves. With the increase in crack depth, the radial magnetic signal increases from −9.1 A/mm to 1.4 A/mm, and the axial magnetic signal increases from 98.93 A/mm to 259.78 A/mm. Both signals exhibit an ascending trend. The slopes of the two curves are 0.95 and 14.62, respectively, indicating that the axial magnetic signal increases more rapidly with the depth of the crack tip. The fitting curves suggest that the radial and axial magnetic signals at the crack tip linearly increase with depth, providing an effective means to assess whether the crack is on the verge of expansion.

3. **The Influence of Crack Axial Length Dz on Magnetic Characteristics**

**Figure 11:**
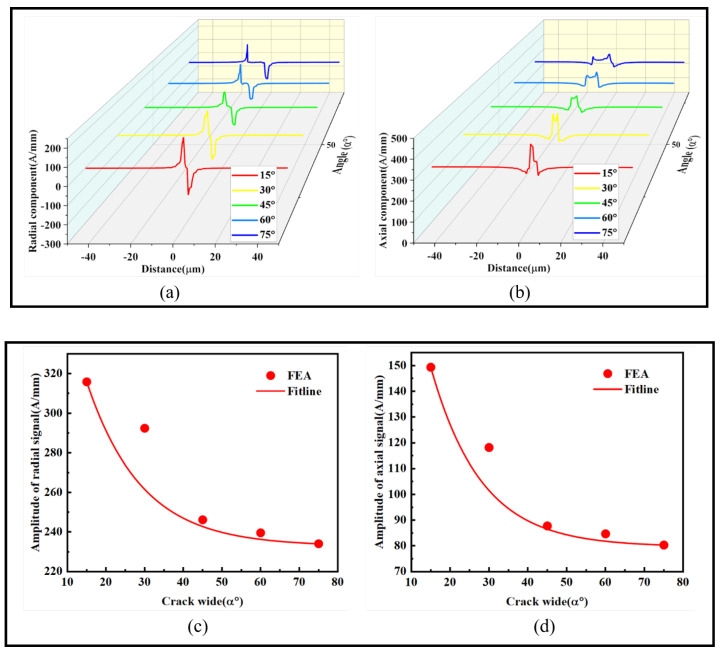
Influence of crack axial length on magnetic signals: (**a**) radial magnetic signal, (**b**) axial magnetic signal, (**c**) radial magnetic signal amplitude difference fitting graph, and (**d**) axial magnetic signal amplitude difference fitting graph.

To study the influence of crack width on the axial and radial magnetic signals, a V-shaped crack with an angle θ of 90° and a depth of 3 μm was set. The *α* angle was increased by 15° at a time, and the amplitude fitting curve of the maximum and minimum values was taken as the influence of crack width on the radial and axial magnetic signals. As shown in Figure 11:

The changes in radial and axial magnetic signals with the increase in angle *α* in Figure 11 show that the peak in the radial magnetic signal gradually shifts towards the direction of the increasing angle, decreasing from 167.73 A/mm to 130.39 A/mm. The direction of the trough remains relatively unchanged with minor fluctuations. In the axial magnetic signal, with the increase in angle, the side with increasing angles shifts from a single peak to a double peak, and the peak value decreases from 378.98 A/mm to 327.93 A/mm. The fitting curves in Figure 11c,d show a nonlinear decrease. The results demonstrate that with the increase in the angle *α*, both radial and axial magnetic signals exhibit a nonlinear decrease. The magnetic signals decrease most rapidly within the range of 30° to 45°, and after 45°, they gradually stabilize. The amplitude distance between radial magnetic signals increases, and axial magnetic signals transition from a single peak to a double peak. This provides insights into the influence of the axial length of the crack on magnetic signals in ferromagnetic microwires.

4. **Influence of Multiple Cracks on Magnetic Characteristics**

**Figure 12:**
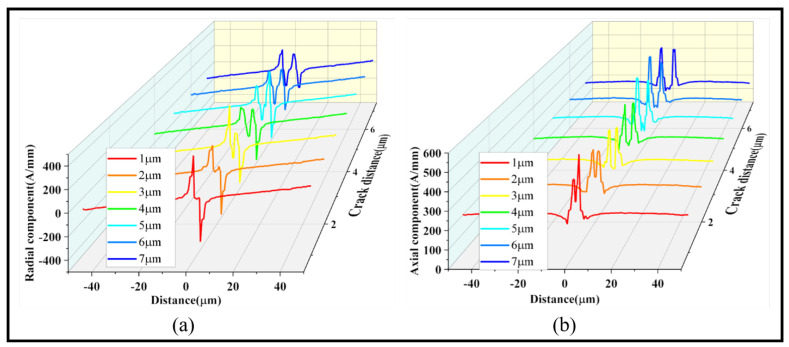
Magnetic signals of two 90° cracks with different spacings: (**a**) radial magnetic signal and (**b**) axial magnetic signal.

During actual service, it is common to have multiple coexisting cracks. To study this phenomenon, cracks of the same size with different spacing were set up to reflect the variation patterns of axial and radial magnetic signals. Figure 12 shows 90° cracks with depths of 3 μm, spaced at 1–7 μm with 1 μm intervals, symmetrically positioned in the center of the ferromagnetic microwires.

According to Figure 12, as the distance between the two cracks increases, both radial and axial magnetic signals transition from a single pattern to two complete patterns. During this transition, the radial magnetic signal rises from the central part, and the axial magnetic signal dips from the central part until the distance reaches 6 μm. After that, the radial magnetic signal has two peaks at 178.33 A/mm and 187.42 A/mm, with two troughs at −209.39 A/mm and −200.61 A/mm. The axial magnetic signal has two peaks at 480.26 A/mm and 472.19 A/mm, with a relative error of around 5%. The formation of two complete patterns indicates that the detection of magnetic signals becomes more intuitive when the distance between the two cracks reaches 6 μm.

5. **Impact of the External Magnetic Field on Magnetic Signals**

**Figure 13:**
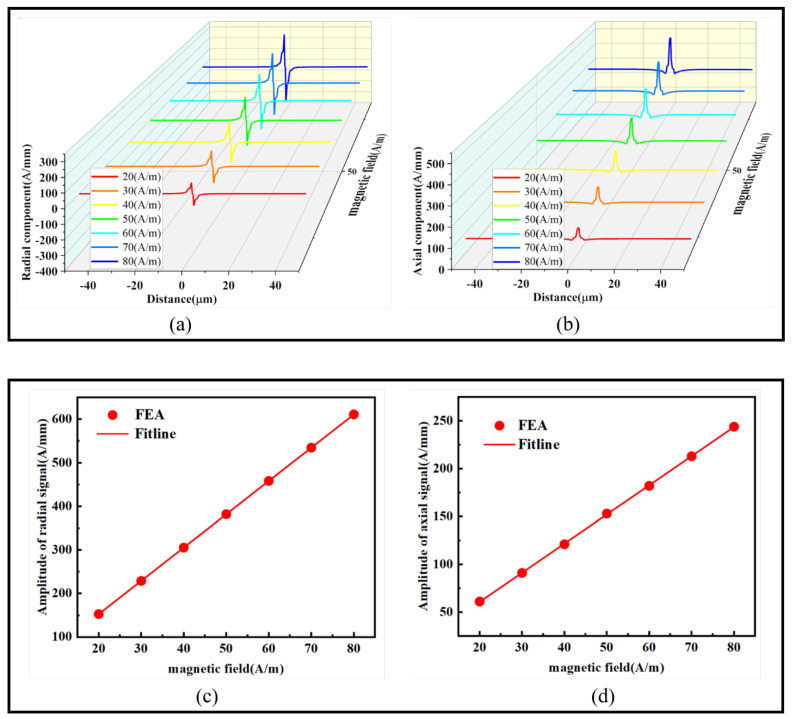
Magnetic signals at the crack under different external magnetic fields: (**a**) radial magnetic signal, (**b**) axial magnetic signal, (**c**) radial magnetic signal amplitude difference fitting graph, and (**d**) axial magnetic signal amplitude difference fitting graph.

To study the influence of the external magnetic field on magnetic signals, an external magnetic field was applied along the positive Z-axis to the 90° crack with a width of 1 μm and a depth of 3 μm. The applied field ranged from 20 to 80 A/m with a 10 A/m interval. This reflected the impact of the crack’s radial and axial magnetic signals under different external magnetic field strengths. The maximum and minimum values of each curve were then used to generate fitting curves, representing the variation of the magnetic field. The results are shown in Figure 13:

As shown in Figure 13a, the peak value of the radial magnetic signal increases from 73.57 A/mm to 294.28 A/mm with the increase in the external magnetic field, while the valley value increases from 79.19 A/mm to 316.76 A/mm. In Figure 13b, the axial magnetic signal peak increases from 121.61 A/mm to 486.01 A/mm with the increasing external magnetic field. The amplitude fitting curves in Figure 13c,d both exhibit a linear increasing trend with slopes of 7.64 and 3.05, respectively. This demonstrate that the magnetic signals exhibit a completely linear increasing trend with the increase in the external magnetic field. Moreover, the radial magnetic signal changes more rapidly with the increasing external magnetic field, making it more sensitive to variations in the external magnetic field.

## 5. Conclusions

We built a numerical model of magnetic charge theory based on the interaction between stress in ferromagnetic microwires and the external magnetic field. Through finite element analysis, it was determined that with an increase in tensile stress on ferromagnetic microwires, the stress concentration effect at the crack tip was maximized, leading to the initiation and propagation of cracks. Subsequently, an analysis of magnetic signal variations at the crack location under different parameter conditions was conducted, characterizing the extent of damage to ferromagnetic microwires during their operational lifespan. The results revealed that an increase in the angle between the external magnetic field and the crack resulted in a linear increase in the magnetic signal. Additionally, due to reduced contact between the measurement point and the crack, the distance between the peak and valley of the radial magnetic signal shortened. Simultaneously, the axial magnetic signal transitioned from a double-peak to a single-peak configuration. As the crack depth increased, the magnetic signal exhibited a linear increase, with fitting function slopes of 50.9 and 41.9 indicating heightened sensitivity and faster changes as the crack depth increased. The magnetic signal at the crack tip also linearly increased. Comparing the slopes of the radial and axial directions, 0.95 and 14.62, respectively, highlighted that the axial magnetic signal underwent faster changes. An increase in crack width led to a non-linear decrease in both radial and axial magnetic signals, with the fastest decline occurring in the 30°–45° range, stabilizing after 45°. The patterns of radial and axial magnetic signals for two different crack spacings transitioned from a single to multiple complete patterns. When the external magnetic field changed, the magnetic signals exhibited a completely linear increasing trend, with peak values showing regular growth. In summary, magnetic signals exhibit noticeable fluctuations only when passing through defects. Therefore, by computationally analyzing the magnitudes of radial and axial magnetic signals under different parameter conditions, it is possible to effectively predict the size, location, and potential re-expansion of cracks in ferromagnetic microwires. This enables the assessment of the degree of damage in ferromagnetic microwires, providing data support for determining whether they can continue in service and for evaluating the overall health status of composite materials.

## Figures and Tables

**Table 1:** Material Parameters of Ferromagnetic Microwires.

Name	Numeric Value	Name	Numeric Value
Radius (D)	22 μm	Length (L)	250 μm
Young’s modulus (E)	134 GPa	Tensile strength ($\sigma_{max}$)	3 GPa
Poisson’s ratio (v)	0.33	Density (ρ)	7720 kg/m^3^

## Data Availability

Data are contained within the article.
